# Supplementary material for: Causal interplay between lactose intolerance and gut microbiota: a combined bidirectional Mendelian randomization and in vivo validation study
Source: Front Nutr. 2026 Jun 1;13:1803337. doi: 10.3389/fnut.2026.1803337 (PMC13265576; doi:10.3389/fnut.2026.1803337)
Supplement: Supplementary file 1 [file Data_Sheet_1.zip › supplementary materials/Forward/forest plot/ebi-a-GCST90027727.finngen_R12_E4_LACTONAS.pdf]

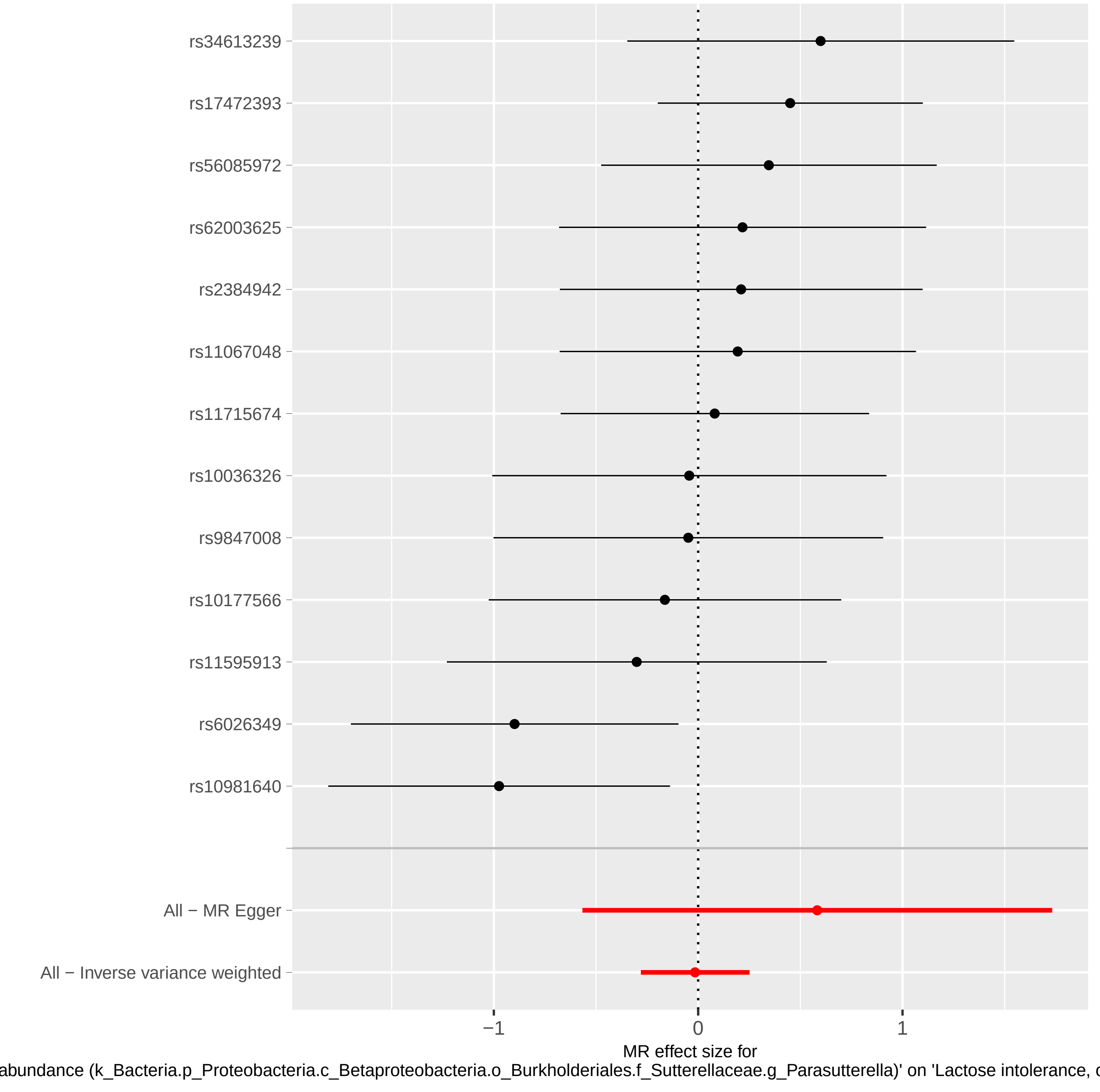

rs34613239
rs17472393
rs56085972
rs62003625
rs2384942
rs11067048
rs11715674
rs10036326
rs9847008
rs10177566
rs11595913
rs6026349
rs10981640
All − MR Egger
All − Inverse variance weighted
−1
0
1
MR effect size for
abundance (k_Bacteria.p_Proteobacteria.c_Betaproteobacteria.o_Burkholderiales.f_Sutterellaceae.g_Parasutterella)' on 'Lactose intolerance, c
